# Lipid Profile Features and Their Associations With Disease Severity and Mortality in Patients With COVID-19

**DOI:** 10.3389/fcvm.2020.584987

**Published:** 2020-12-04

**Authors:** Jia Teng Sun, Zhongli Chen, Peng Nie, Heng Ge, Long Shen, Fan Yang, Xiao Long Qu, Xiao Ying Ying, Yong Zhou, Wei Wang, Min Zhang, Jun Pu

**Affiliations:** ^1^Division of Cardiology, Renji Hospital, Shanghai Jiao Tong University School of Medicine, Shanghai, China; ^2^Division of Pulmonary and Critical Care Medicine, Leishenshan Hospital, Wuhan, China; ^3^Institute of Cardiovascular Disease, Ruijin Hospital, Shanghai Jiao Tong University School of Medicine, Shanghai, China

**Keywords:** HDL-C, apoA-1, inflammation, lipid, COVID-19

## Abstract

**Background:** Emerging studies have described and analyzed epidemiological, clinical, laboratory, and radiological features of COVID-19 patients. Yet, scarce information is available regarding the association of lipid profile features and disease severity and mortality.

**Methods:** We conducted a prospective observational cohort study to investigate lipid profile features in patients with COVID-19. From 9 February to 4 April 2020, a total of 99 patients (31 critically ill and 20 severely ill) with confirmed COVID-19 were included in the study. Dynamic alterations in lipid profiles were recorded and tracked. Outcomes were followed up until 4 April 2020.

**Results:** We found that high-density lipoprotein-cholesterol (HDL-C) and apolipoprotein A-1 (apoA-1) levels were significantly lower in the severe disease group, with mortality cases showing the lowest levels (*p* < 0.0001). Furthermore, HDL-C and apoA-1 levels were independently associated with disease severity (apoA-1: odds ratio (OR): 0.651, 95% confidence interval (CI): 0.456–0.929, *p* = 0.018; HDL-C: OR: 0.643, 95% CI: 0.456–0.906, *p* = 0.012). For predicting disease severity, the areas under the receiver operating characteristic curves (AUCs) of HDL-C and apoA-1 levels at admission were 0.78 (95% CI, 0.70–0.85) and 0.85 (95% CI, 0.76–0.91), respectively. For in-hospital deaths, HDL-C and apoA-1 levels demonstrated similar discrimination ability, with AUCs of 0.75 (95% CI, 0.61–0.88) and 0.74 (95% CI, 0.61–0.88), respectively. Moreover, patients with lower serum concentrations of apoA-1 (<0.95 g/L) or HDL-C (<0.84 mmol/l) had higher mortality rates during hospitalization (log-rank *p* < 0.001). Notably, levels of apoA-1 and HDL-C were inversely proportional to disease severity. The survivors of severe cases showed significant recovery of apoA-1 levels at the end of hospitalization (vs. midterm apoA-1 levels, *p* = 0.02), whereas the mortality cases demonstrated continuously lower apoA-1 levels throughout hospitalization. Correlation analysis revealed that apoA-1 and HDL-C levels were negatively correlated with both admission levels and highest concentrations of C-reactive protein and interleukin-6.

**Conclusions:** Severely ill COVID-19 patients featured low HDL-C and apoA-1 levels, which were strongly correlated with inflammatory states. Thus, low apoA-1 and HDL-C levels may be promising predictors for severe disease and in-hospital mortality in patients suffering from COVID-19.

## Introduction

As Coronavirus Disease 2019 (COVID-19) continues to spread worldwide, millions of people across hundreds of countries have been impacted. Epidemiological data show that although most cases are mild, severely ill patients rapidly progress to acute respiratory disease, multi-organ failure, and septic shock, with a remarkably increased mortality rate. Therefore, early identification of risk factors for COVID-19 severity and progression is of great importance.

Mounting evidence suggests that an impaired immune function and hyper-inflammatory response are characteristics of COVID-19 severity and mortality (1–3). Systemic inflammation and sepsis are prevalent metabolic disorders accompanying severe COVID-19 (4). Furthermore, proteome analysis suggests that patients with severe COVID-19 display dysregulated lipid metabolism (5). Dyslipidemia is associated with damage to the immune, respiratory, and cardiovascular systems, along with high levels of proinflammatory cytokines. Furthermore, dyslipidemia is casually associated with increased risk of thrombotic complications, endothelial dysfunction, and higher platelet activity (6). Thus, lipid dysregulation may contribute to morbidity and mortality from COVID-19 infection. However, the characteristics and dynamic changes in lipid profiles in COVID-19 patients, as well as their predictive value in disease severity and mortality, remain largely unknown.

Here, we performed an observational cohort study to investigate the lipid profile features of patients with COVID-19 and illuminate the associations between lipid features and disease severity/mortality.

## Materials and Methods

### Study Population

This observational cohort study prospectively included 99 COVID-19-confirmed inpatients treated from 9 February to 4 April 2020 in Leishenshan Hospital, an urgently constructed hospital designated for COVID-19 patients located in Wuhan, China. All patients were diagnosed with COVID-19 according to interim guidance provided by the World Health Organization (WHO) (7). COVID-19 severity was classified according to the Guidelines on the Diagnosis and Treatment of COVID-19 released by the National Health Commission of China (version 7). Criteria for severe cases included any of the following: (1) respiratory rate ≥ 30 per min; (2) blood oxygen saturation (SPO_2_) ≤ 93% at rest; (3) partial pressure of arterial oxygen to fraction of inspired oxygen ratio <300; (4) more than 50% of lung infiltrates within 24–48 h; or (5) patients needing mechanical respiratory support or presenting with septic shock or multi-organ dysfunction or failure. All patients had a definite outcome (discharged, continued treatment, deceased) before data analysis.

### Data Collection

Time from symptom onset to hospitalization and length of hospital stay were recorded. All epidemiological, clinical, laboratory, and outcome data were collected with standardized data collection forms from the electronic medical records system at Leishenshan Hospital. Personal history, including comorbidities, was confirmed with patients or family members. For information not available from the electronic medical records, researchers also communicated directly with patients or their families to obtain additional epidemiological and symptom data. Lipid profiles, including total cholesterol (TC), triglycerides (TG), low-density lipoprotein-cholesterol (LDL-C), high-density lipoprotein-cholesterol (HDL-C), apolipoprotein A-1 (apoA-1), and apolipoprotein B (apoB), were first determined within 24 h of admission. A subset of patients had multiple lipid and cytokine metrics (i.e., collected more than once); therefore, these data were included for longitudinal analysis. Dynamic alterations in the above indicators were recorded. The Sequential Organ Failure Assessment (SOFA) score (https://www.mdcalc.com/sequential-organ-failure-assessmment-sofa-score) were calculated for each participant on admission. Two researchers independently reviewed the forms to double-check the data collected.

### Outcome Definition

Outcomes were followed up until 4 April 2020. The primary outcome in the study was defined as in-hospital death.

### Statistics Analysis

No preliminary sample size calculation was evaluated, considering the observational nature of our study about this emerging infectious disease. Continuous variables were expressed as medians with interquartile ranges (IQR) and compared using unpaired Student's *t*-test or Mann-Whitney U test. Categorical data were expressed as absolute values and percentages and were compared using chi-square or Fisher's exact tests. Univariate and multivariable analyses were conducted to examine the associations between lipids and disease severity. To assess the discrimination ability of each lipid marker for outcome, receiver operating characteristic (ROC) curves were calculated, and the optimal cutoff values were determined by maximizing the Youden index. Spearman tests were used to analyze the correlations between lipids and inflammatory factors. Survival differences among groups with different lipid concentrations were compared by Kaplan-Meier analysis using the log-rank test. Significance levels were set based on two-sided α < 0.05. Data analyses were performed in statistical packages R (The R Foundation; http://www.r-project.org; version 3.6.1) and SPSS 22.0. Diagrams were plotted by GraphPad Prism 8.0 (GraphPad Software, USA).

## Results

### Baseline Characteristics

A total of 99 laboratory-confirmed COVID-19 patients were prospectively enrolled in this study. As shown in Table 1, the median time from symptom onset to admission was comparable between mild and severe cases [20.00 (IQR: 14.00–26.00) days vs. 19.00 (IQR, 10.25–30.00) days, *p* = 0.841] as well as between severe-surviving and severe-non-surviving groups [20.00 (IQR: 10.50–30.00) days vs. 17.00 (IQR, 10.00–30.00) days, *p* = 0.663]. Compared with mild cases, severely ill patients were older (severe: median 70.5 years: IQR, 61.3–81.8 vs. mild: 52 years: IQR, 42.0–62.0) and more likely to have comorbidities (severe: 84% vs. mild: 59.2%) and higher SOFA scores (severe: median, 5, IQR, 2–7 vs. mild: median, 0, IQR, 0–1). No sex differences were found between the mild and severe groups. Fourteen patients received mechanical ventilation in the severe group, whereas no mechanical ventilation was used in the mild cases. A total of 15 severe group patients died in hospital. Mechanical ventilation was more frequently applied among non-survivors. Severe-non-surviving cases presented significantly higher SOFA scores (median, 8.00, IQR, 7.50–10.00) than severe-surviving cases (median, 3.00, IQR, 1.25–5.00). Statin and antiviral treatment were similar among the groups. However, corticosteroid and antibiotic use differed significantly between severe and mild patients. Of note, more deceased patients received corticosteroid therapy compared with severe-surviving patients. The time from symptom onset to admission was comparable between the mild and severe groups [20 IQR (14–26) days vs. 19 IQR (10.25–30) days, *p* = 0.841] as well as between the severe-surviving and severe-non-surviving groups [20 IQR (10.5–30) days vs. 17 IQR (10–30) days, *p* = 0.663]. Mild patients experienced a longer hospitalization stay compared to severe patients [20 IQR (15–25) days vs. 15 IQR (9–20.5) days, *p* = 0.012]. Length of hospitalization was similar between the severe-surviving and severe-non-surviving groups [15 IQR (9–22.5) days vs. 15 IQR (10–18.5) days, *p* = 0.706].

**Table 1:** Clinical characteristics and laboratory assessments in COVID-19 patients.

	**Mild (*n* = 49)**	**Severe(*n* = 50)**	***p*-value**	**Severe (*****n*** **=** **50)**		***p*-value**
				**Severe-surviving (*n* = 35)**	**Severe-non-surviving (*n* = 15)**	
Age, years	52.00 (42.00–62.00)	70.50 (61.25–80.75)	<0.001	69.00 (61.50–80.50)	73.00 (63.50–78.50)	0.695
Male, *n*%	26 (53.06%)	34 (68.00%)	0.128	26 (74.29%)	8 (53.33%)	0.191
SOFA score	0 (0–1)	5.0 (2.0–7.0)	<0.001	3.00 (1.25–5.00)	8.00 (7.50–10.00)	<0.001
Mechanical ventilation, n%	0 (0.00%)	15 (30.00%)	<0.001	5 (14.29%)	10 (66.67%)	<0.001
Symptom to admission duration, days	20.00 (14.00–26.00)	19.00 (10.25–30.00)	0.841	20.00 (10.50–30.00)	17.00 (10.00–30.00)	0.663
Length of hospitalization, days	20.00 (15.00–25.00)	15.00 (9.00–20.50)	0.012	15.00 (9.00–22.50)	15.00 (10.00–18.50)	0.706
**Symptom**						
- Fever, *n*%	38 (77.55%)	28 (56.0%)	0.023	20 (57.14%)	8 (53.33%)	0.804
- Diarrhea, *n*%	9 (18.37%)	6 (12.0%)	0.377	6 (17.14%)	0 (0.00%)	0.160
- Fatigue, *n*%	13 (26.53%)	19 (38.0%)	0.222	16 (45.71%)	3 (20.00%)	0.117
- Cough, *n*%	29 (59.18%)	26 (52.0%)	0.472	19 (54.29%)	7 (46.67%)	0.760
- Chest pain, *n*%	19 (38.78%)	23 (46.0%)	0.467	18 (51.43%)	5 (33.33%)	0.355
- Dyspnea, *n*%	13 (26.53%)	24 (48.0%)	0.027	21 (60.00%)	3 (20.00%)	0.014
**Comorbidities**, ***n*****%**	29 (59.18%)	42 (84.0%)	0.006	27 (77.14%)	15 (100.00%)	0.086
- Diabetes, *n*%	7 (14.29%)	24 (48.00%)	<0.001	16 (45.71%)	7 (46.67%)	1.000
- Hypertension, *n*%	18 (36.73%)	28 (56.00%)	0.085	19 (54.29%)	9 (60.00%)	0.765
- Pulmonary disease, *n*%	5 (10.20%)	6 (12.00%)	0.563	4 (11.43%)	2 (13.33%)	0.849
- Heart failure, *n*%	3 (6.12%)	14 (26.00%)	0.007	11 (31.43%)	3 (20.00%)	0.507
- CKD, *n*%	0 (0.00%)	16 (32.00%)	<0.001	11 (31.43%)	5 (33.33%)	1.000
- CAD, *n*%	1 (2.04%)	13 (26.00%)	<0.001	10 (28.57%)	3 (20.00%)	0.728
- Tumor, *n*%	3 (6.12%)	4 (8.00%)	0.716	2 (5.71%)	2 (13.33%)	0.574
- Autoimmune disease, *n*%	0 (0.00%)	2 (4.00%)	0.157	1 (2.86%)	1 (6.67%)	0.514
- Dyslipidemia, *n*%	4 (8.16%)	8 (16.00%)	0.147	4 (11.43%)	4 (26.67%)	0.178
**Laboratory findings**						
- Leukocytes ×10^9^/L	5.68 (4.67–7.02)	7.42 (5.27–10.41)	<0.001	7.33 (5.68–9.68)	9.69 (5.00–14.82)	0.403
- Neutrophil ×10^9^/L	3.04 (2.61–3.94)	6.01 (3.96–8.91)	<0.001	5.64 (3.96–7.28)	8.00 (4.26–11.82)	0.182
- Lymphocyte ×10^9^/L	1.66 (1.04–2.26)	0.83 (0.67–1.24)	<0.001	0.90 (0.71–1.33)	0.70 (0.28–0.89)	0.020
- Platelets ×10^9^/L	199.00 (171.00–256.00)	199.00 (133.75–274.50)	0.378	216.00 (174.00–281.00)	117.00 (80.50–152.50)	0.003
- Erythrocytes ×10^12^/L	4.13 (3.87–4.51)	3.35 (2.83–3.80)	<0.001	3.31 (2.88–3.77)	3.39 (2.55–3.74)	0.594
- Hemoglobin, g/L	128.00 (119.00–137.00)	103.00 (84.00–117.50)	<0.001	105.0–3.74 (84.50–120.00)	101.00 (84.00–112.00)	0.775
- CRP, mg/L	0.81 (0.52–2.61)	33.91 (9.14–82.47)	<0.001	22.66 (6.26–63.94)	69.53 (30.16–114.89)	0.014
- Procalcitonin, ng/mL	0.03 (0.02–0.04)	0.32 (0.09–1.04)	<0.001	0.16 (0.09–0.52)	0.87 (0.44–1.53)	0.017
- ESR, mm/H	12.00 (7.00–23.00)	43.00 (21.25–60.75)	<0.001	42.00 (21.50–59.50)	44.00 (17.00–67.50)	0.916
- SAA, mg/L	5.00 (5.00–5.30)	54.78 (13.61–214.33)	<0.001	35.44 (9.61–244.24)	102 (32.4–270.46)	0.016
- PT, s	11.40 (10.90–11.70)	12.10 (11.43–13.55)	<0.001	12.10 (11.35–13.60)	12.10 (11.55–14.40)	0.491
- INR	0.98 (0.93–1.01)	1.05 (0.98–1.18)	<0.001	1.05 (0.97–1.19)	1.05 (0.99–1.27)	0.484
- Fibrinogen, g/L	2.66 (2.40–2.95)	4.04 (3.21–5.60)	<0.001	3.99 (3.24–5.72)	4.75 (3.25–5.60)	0.832
- D-Dimer, mg/L	0.29 (0.15–0.59)	2.94 (1.64–4.09)	<0.001	2.31 (1.45–3.74)	4.03 (2.57–6.42)	0.088
- BNP, pg/mL	7.00 (6.00–13.87)	117.66 (28.15–342.00)	<0.001	119.79 (26.27–593.00)	115.22 (33.84–189.34)	0.695
- Hs-cTnI, ng/ml	0.01 (0.01–0.01)	0.03 (0.01–0.06)	<0.001	0.02 (0.01–0.06)	0.03 (0.03–0.06)	0.078
- ALT, μ/L	28.00 (19.00–42.00)	21.00 (12.50–29.50)	0.059	21.00 (13.00–27.00)	24.00 (16.50–36.50)	0.532
- AST,μ/L	20.00 (17.00–26.00)	24.00 (18.00–32.75)	0.053	22.00 (18.00–31.00)	28.00 (18.50–44.00)	0.385
- Albumin, g/L	38.10 (36.10–41.30)	30.50 (28.40–35.68)	<0.001	30.50 (28.80–34.55)	29.40 (25.10–34.90)	0.346
- TBIL, μmol/L	9.24 (7.40–12.70)	9.40 (6.55–14.10)	0.607	8.40 (6.35–11.65)	14.10 (7.25–18.10)	0.159
- Glucose, mmol/L	4.69 (4.38–5.03)	5.97 (4.88–8.20)	<0.001	5.73 (4.89–7.48)	6.69 (4.62–12.05)	0.498
- BUN, mmol/L	4.70 (4.00–5.30)	8.70 (5.32–15.60)	<0.001	7.20 (4.60–11.05)	14.40 (8.80–37.40)	0.026
- Creatinine, μmol/L	60.20 (50.70–70.40)	82.20 (56.73–154.83)	<0.001	75.00 (56.25–108.45)	98.20 (68.20–235.20)	0.295
- Total cholesterol, mmol/L	4.52 (3.63–4.9)	3.51 (2.90–4.48)	<0.001	3.59 (2.98–4.48)	3.18 (2.58–4.25)	0.553
- Triglycerides, mmol/L	1.21 (0.81–1.80)	0.96 (0.70–1.62)	0.114	0.90 (0.70–1.38)	1.00 (0.82–2.71)	0.010
- LDL-C, mmol/L	2.57 (2.04–2.96)	2.16 (1.58–2.68)	0.016	2.19 (1.64–2.83)	1.76 (1.49–2.64)	0.494
- HDL-C, mmol/L	1.18 (1.00–1.42)	0.94 (0.74–1.12)	<0.001	0.97 (0.76–1.08)	0.77 (0.61–0.99)	0.112
- apoA-1, g/L	1.42 (1.22–1.64)	1.01 (0.79–1.23)	<0.001	1.03 (0.80–1.25)	0.84 (0.64–1.19)	0.277
- apoB, g/L	0.93 (0.79–1.08)	0.80 (0.69–1.14)	0.205	0.86 (0.73–1.14)	0.70 (0.66–1.07)	0.277
- IL.6, pg/mL	1.29 (0.75–3.37)	38.45 (12.59–80.07)	<0.001	23.84 (10.55–41.88)	124.90 (58.45–241.45)	<0.001
- IL.1β, pg/mL	3.00 (2.00–3.29)	3.75 (3.00–5.00)	0.009	3.00 (3.00–4.07)	5.00 (3.67–6.32)	0.023
- IL.8, pg/mL	6.00 (3.80–8.60)	16.70 (13.00–27.80)	<0.001	16.00 (11.50–22.00)	28.40 (19.50–49.00)	0.005
- IL.10, pg/mL	3.00 (2.00–3.56)	4.01 (3.00–8.97)	<0.001	4.00 (3.00–7.55)	8.20 (3.43–15.00)	0.146
- IL2R, U/mL	0.31 (0.22–0.43)	0.81 (0.57–1.65)	<0.001	0.72 (0.58–1.42)	1.56 (0.60–2.94)	0.147
- TNF α, pg/mL	6.50 (5.50–7.16)	10.61 (7.75–14.73)	<0.001	10.70 (7.45–14.38)	11.50 (8.50–19.45)	0.427
**Treatment**, ***n*****%**						
Antibiotic therapy	17 (34.70%)	50 (100%)	<0.001	35 (100%)	15 (100%)	–
Antiviral therapy	47 (95.92%)	48 (96.00%)	0.984	34 (97.14%)	14 (93.33%)	0.529
Use of corticosteroids	0 (0%)	19 (38.00%)	<0.001	10 (28.57%)	9 (60.00%)	0.036
Statin	8 (16.32%)	15 (30.00%)	0.107	11 (31.42%)	4 (26.67%)	0.736

*Categorical data are expressed as absolute values and percentages and were compared using chi-square or Fisher exact tests. Continuous variables were expressed as medians with interquartile ranges (IQR) and compared by unpaired Student's t-test or Mann-Whitney U test. AST, aspartate aminotransferase; ALT,alanine aminotransferase; BUN, blood urea nitrogen; BNP, brain natriuretic peptide; Hs-cTnI, hypersensitive troponin I;CRP, C-reactive protein; CKD, chronic kidney disease; CAD, coronary artery disease; ESR, erythrocyte sedimentation rate; SAA, serum amyloid A; IL, interleukin; IL2R, interleukin2 receptor; LDL-C, low-densitylipoprotein-cholesterol; HDL-C, high-density lipoprotein-cholesterol; apoA-1, apolipoproteinA-1; apoB, apolipoproteinB; INR,international standard ratio; PT, prothrombin time; TNF α, tumor necrosis factorα; TBIL, total bilirubin; SOFA, Sequential Organ Failure Assessment*.

### Laboratory Parameters and Lipid Variation on Admission

**Figure 1:**
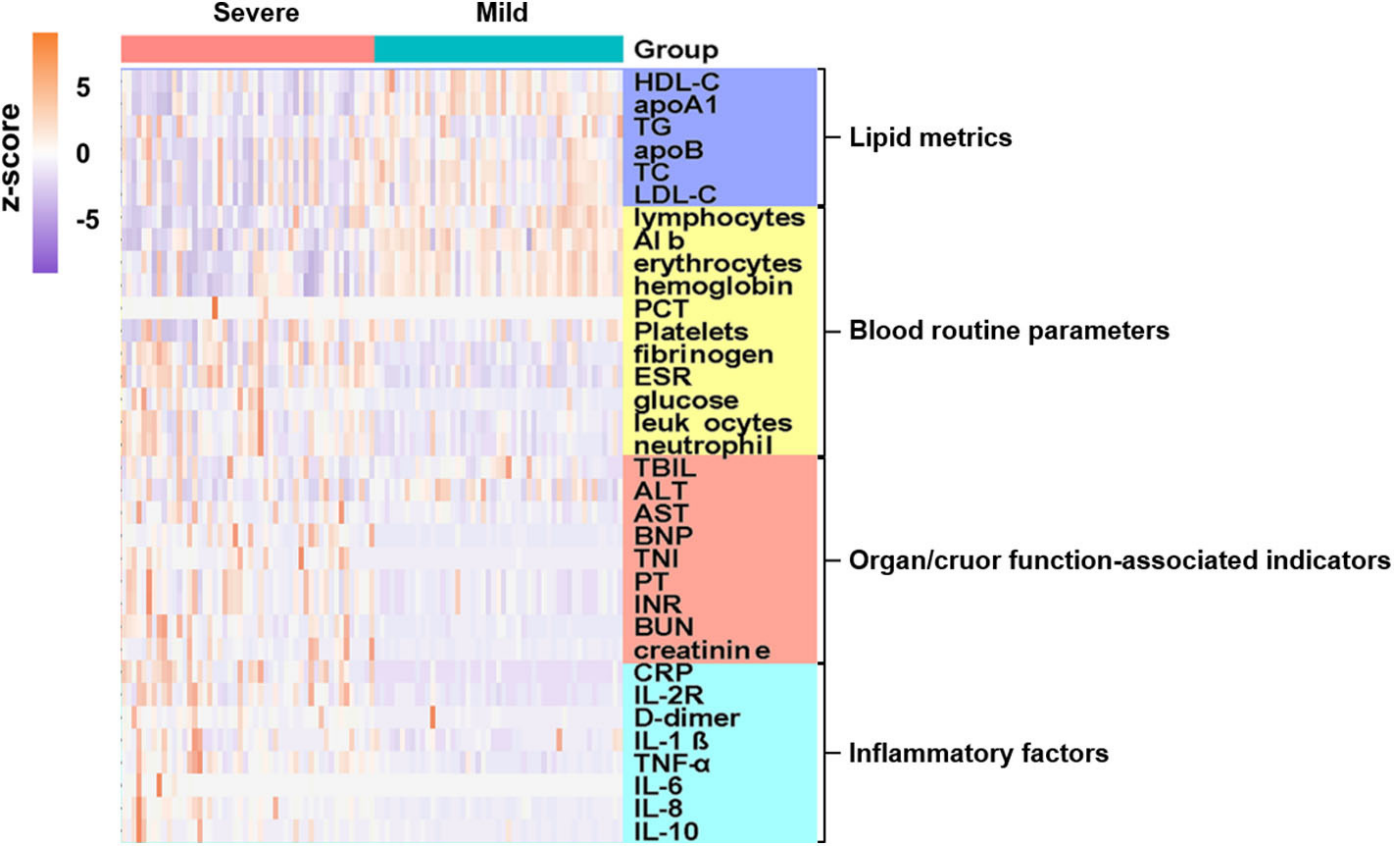
Admission characteristics of laboratory parameters between mild and severe COVID-19 patients. Hierarchical clustering was applied based on laboratory parameters. Heatmap indicates enriched concentration of laboratory indicators in mild and severe cases. Levels of laboratory metrics were scaled by calculating z-scores (subtracting mean, then dividing by standard deviation of each row). Laboratory metrics were categorized into four major groups, i.e., lipid metrics, routine blood parameters, organ/cruor function-associated indicators, and inflammatory factors, with color bars on right side of plot indicating each analyte category. Y-axis represents laboratory values after z-scoring by row; x-axis represents individual cases. Annotations show severe cases in pink and mild cases in cyan.

For major laboratory characteristics, mild and severe COVID-19 cases demonstrated significant deviation in terms of blood cell proportions, coagulation functions, cardiac and renal functions, inflammatory indicators, and lipid profiles. Hierarchical clustering was performed to visualize the differences in laboratory parameters between mild and severe COVID-19 patients. The resulting heatmap illustrated different enrichment in blood indicators between mild and severe cases (Figure 1, Supplementary Figure 1). Notably, inflammatory cytokines, which are organ injury-associated indicators, were found at higher concentrations in the severe cases, whereas certain blood indicators, including lymphocytes, erythrocytes, hemoglobin, and albumin, were higher in the mild group.

**Figure 2:**
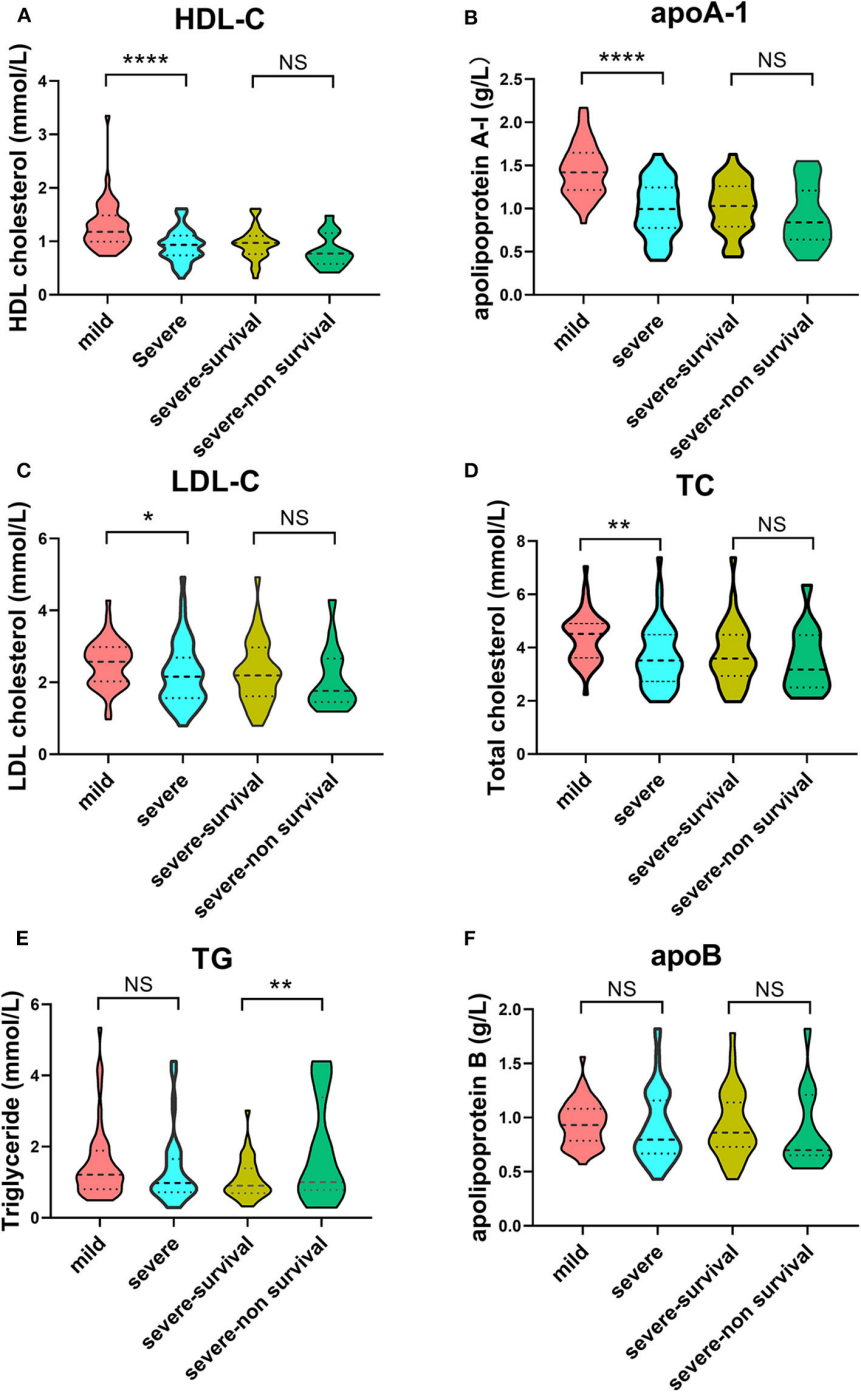
Violin plots of lipid features of mild vs. severe and severe survivors vs. severe non-survivors. Plots demonstrate lipid concentration within each group. Horizontal dotted lines represent first and third quartiles; horizontal dashed lines within plot indicate median of lipid levels. Dunnett's test was applied to assess significance of differences with mild cases serving as the control. (^****^p < 0.0001, ^**^p < 0.01, ^*^p < 0.05).

In terms of lipid profiles, we detected lower concentrations of HDL-C, apoA-1, LDL-C, and TC in the severe group compared with the mild group (Figures 2A–D). The TG level was significantly increased in the severe-non-surviving cases compared with the severe-surviving cases (Figure 2E), while HDL-C, apoA-1, LDL-C, TC and apoB concentrations were comparable between these two groups (Figures 2A–D,F).

### Lipid Profiles and Risk of Severe Condition

Based on the distinct lipid profile features between the severe and mild cases, we performed univariate and multivariate logistic regression analyses to explore the associations between lipid concentrations and disease severity. According to univariate analysis, TC, HDL-C, and apoA-1 levels were associated with severe disease as both continuous and categorical variables (divided by tertiles), whereas LDL-C and TG did not reach statistical significance. Remarkably, based on multivariate analysis, we found that apoA-1 (OR: 0.651 95% CI: 0.456–0.929, *p* = 0.018) and HDL-C (OR: 0.643 95% CI: 0.456–0.906, *p* = 0.012) were still independently associated with severity after adjusting for well-recognized risk factors: i.e., age and albumin, D-dimer, C-reactive protein (CRP), and interleukin-6 (IL-6) levels (Table 2). Moreover, patients with the highest tertile of HDL-C and apoA-1 displayed the lowest risk for severe COVID-19. Even after considering comorbidities and SOFA scores for further adjustment, apoA-1 and HDL-C levels remained independently associated with severe status of the disease (Supplementary Table 1). The ROC curves confirmed the significant predictive value of HDL-C and apoA-1 for the presence of severe cases. As shown in Table 3, apoA-1 ≤ 1.16 g/L predicted severity with a specificity of 0.86, sensitivity of 0.66, and area under ROC curve (AUC) of 0.85 (95% CI: 0.76–0.91; *p* < 0.001). An optimal serum HDL-C cut-off of 1.00 mmol/L provided diagnostic specificity and sensitivity of 75.5 and 68.2%, respectively, for severe cases. TC also displayed prognostic capability, but LDL-C, apoB, and TG showed weak discrimination of the severe condition.

**Table 2:** Logistic regression analysis for severity in COVID-19 patients.

	**Univariate OR (95% CI)**	***p*-value**	Adjusted OR^*^ (95% CI)	***p*-value**
**apoA-1 (10**^**−1**^**g/L)**	0.617 (0.507, 0.751)	<0.001	0.651 (0.456, 0.929)	0.018
**apoA-1 group**				
Q1 (4–10.4)	Ref		Ref	
Q2 (10.5–13.8)	0.126 (0.036, 0.443)	0.001	0.538 (0.059, 4.882)	0.581
Q3 (14.0–21.7)	0.036 (0.009, 0.136)	<0.001	0.066 (0.005, 0.823)	0.034
apoA-1 group trend		<0.001		0.023
**HDL-C (10**^**−1**^**mmol/L)**	0.709 (0.602, 0.835)	<0.001	0.643 (0.456, 0.906)	0.012
**HDL-C group**				
Q1 (3.1–9.1)	Ref		Ref	
Q2 (9.3–11.78)	0.400 (0.139, 1.147)	0.090	0.264 (0.028, 2.469)	0.242
Q3 (11.8–33.5)	0.103 (0.033, 0.316)	<0.001	0.065 (0.005, 0.778) 0.03093	0.031
HDL-C group trend		<0.001		0.029
**Total cholesterol (mmol/L)**	0.505 (0.328, 0.776)	0.002	0.866 (0.425, 1.766)	0.693
**TC group**				
Q1 (1.97–3.44)	Ref		Ref	
Q2 (3.49–4.54)	0.301 (0.106, 0.860)	0.025	0.497 (0.079, 3.146)	0.458
Q3 (4.56–7.38)	0.120 (0.040, 0.362)	<0.001	0.338 (0.048, 2.360)	0.274
TC group trend		<0.001		0.281
**Triglycerides (mmol/L)**	0.808 (0.538, 1.214)	0.304	0.808 (0.269, 2.423)	0.703
**TG group**				
Q1 (0.29–0.82)	Ref		Ref	
Q2 (0.84–1.43)	0.778 (0.295, 2.051)	0.611	1.212 (0.242, 6.079)	0.815
Q3 (1.44–5.35)	0.648 (0.244, 1.724)	0.385	1.006 (0.119, 8.472)	0.996
TG group trend		0.402		0.993
**LDL-C (mmol/L)**	0.588 (0.343, 1.007)	0.053	1.281 (0.508, 3.230)	0.599
**LDL-C group**				
Q1 (0.79–1.96)	Ref		Ref	
Q2 (2.01–2.68)	0.471 (0.174, 1.273)	0.137	1.614 (0.208, 12.524)	0.647
Q3 (2.70–4.93)	0.286 (0.104, 0.787)	0.015	1.709 (0.241, 12.144)	0.592
LDL-C group trend		0.01545		0.62094
**apoB (g/L)**	0.638 (0.147, 2.766)	0.548	3.908 (0.279, 54.710)	0.311
**apoB group**				
Q1 (0.43–0.76)	Ref		Ref	
Q2 (0.77–1.02)	0.300 (0.108, 0.830)	0.02	1.022 (0.147, 7.105)	0.982
Q3 (1.03–1.82)	0.444 (0.165, 1.194)	0.108	1.706 (0.270, 10.797)	0.57
apoB group trend		0.145		0.512

*Logistic regression was used to determine association between lipid profile with severity of COVID-19*.

* *Adjusted for age and albumin, D-dimer, CRP, and IL-6 levels. LDL-C, low-densitylipoprotein-cholesterol; HDL-C, high-density lipoprotein-cholesterol; apoA-1, apolipoproteinA-1; apoB, apolipoprotein B; TG, triglycerides; TC, total cholesterol*.

**Table 3:** Diagnostic values of lipid profiles in assessment of COVID-19 severity.

	**AUC (95% CI)**	**Best threshold**	**Specificity**	**Sensitivity**	***p-*value**
apoA-1	0.85 (0.76–0.91)	1.16	0.86	0.66	<0.001
HDL-C	0.78 (0.69–0.85)	1.00	0.76	0.68	<0.001
TC	0.71 (0.61–0.81)	3.24	0.94	0.42	<0.001
apoB	0.58 (0.46–0.68)	0.78	0.78	0.46	0.192
LDL-C	0.62 (0.52–0.76)	1.78	0.92	0.40	0.016
TG	0.59 (0.46–0.70)	1.13	0.61	0.62	0.126
apoA-1 + HDL-C	0.85 (0.77–0.92)	–	0.86	0.66	<0.001

*AUC, area under the curve; LDL-C, low-density lipoprotein-cholesterol; HDL-C, high-density lipoprotein-cholesterol; apoA-1, apolipoprotein A-1; apoB, apolipoprotein B; TG, triglycerides; TC, total cholesterol*.

### Association of Lipid Biomarkers With COVID-19 Mortality

**Figure 3:**
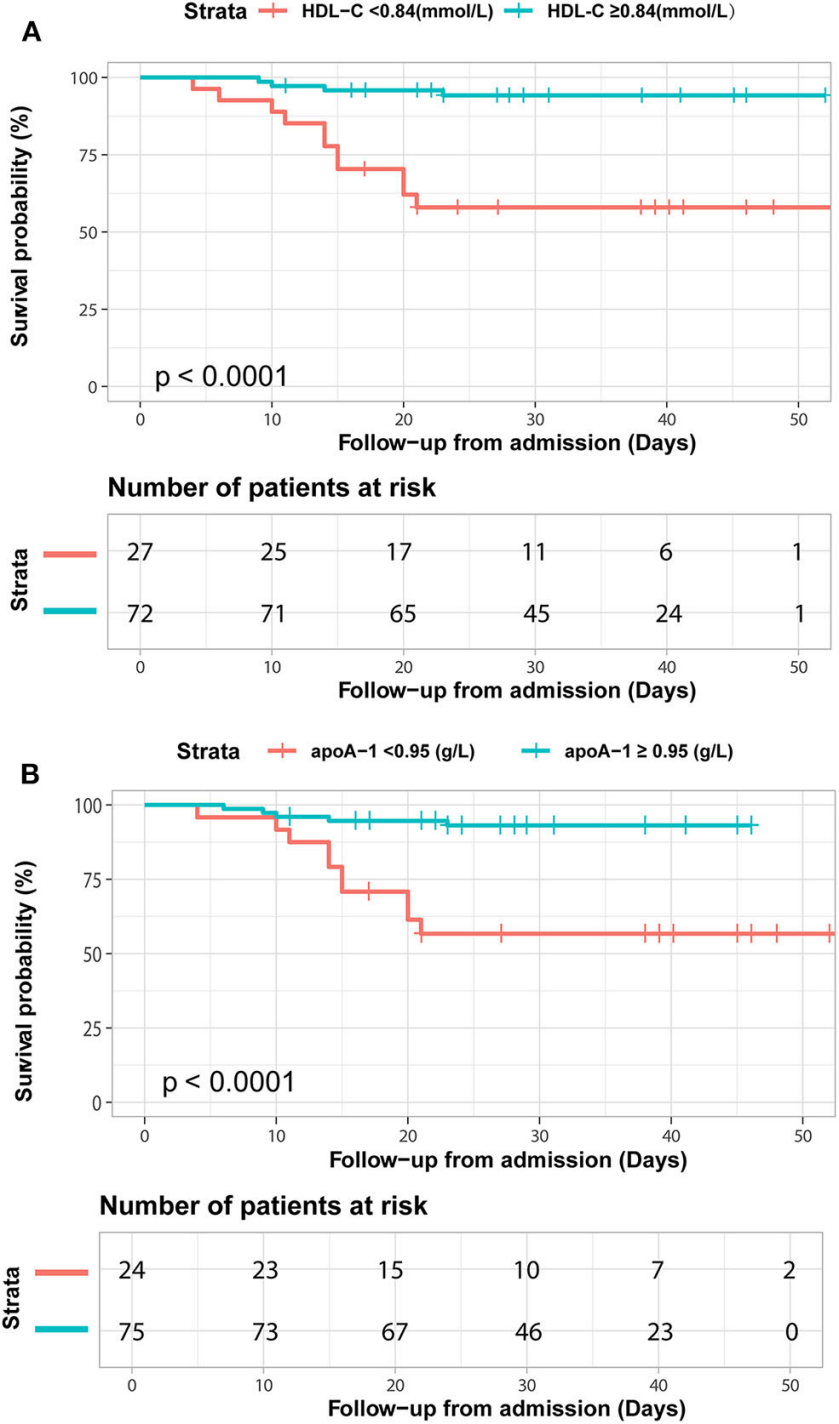
Kaplan-Meier survival curves for in-hospital deaths based on dichotomized HDL-C and apoA-1 concentrations. COVID-19 patients with apoA-1 **(A)** and HDL-C **(B)** levels above and below the optimal cutoff value (calculated by ROC analysis) showed obvious disparity in survival time (*p* < 0.0001).

We further detected the predictive performance of lipid profiles for in-hospital death. Notably, ROC analysis revealed that HDL-C and apoA-1 remained valuable for predicting in-hospital death. At a threshold of 0.95 g/L, the AUC of the ROC curve of apoA-1 for death was 0.74 (95% CI 0.61–0.88, *p* = 0.002). With a cut-off of 0.84 mmol/L, the AUC of HDL-C for death was 0.75 (95% CI: 0.61–0.88, *p* = 0.002) (Table 4). Moreover, the Kaplan-Meier survival curves and log-rank tests demonstrated that patients with lower apoA-1 or HDL-C levels had a higher rate of in-hospital mortality (divided according to the best threshold) (Figure 3).

**Table 4:** Diagnostic values of lipid profiles in assessment of COVID-19 mortality.

	**AUC (95% CI)**	**Best threshold**	**Specificity**	**Sensitivity**	***p*-value**
apoA-1	0.74 (0.61–0.88)	0.95	0.83	0.67	0.002
HDL-C	0.75 (0.61–0.88)	0.84	0.81	0.73	0.002
apoB	0.62 (0.43–0.79)	0.71	0.85	0.53	0.093
LDL-C	0.64 (0.46–0.80)	1.83	0.80	0.60	0.054
TG	0.44 (0.27–0.61)	1.01	0.58	0.53	0.444
TC	0.66 (0.51–0.80)	3.18	0.83	0.53	0.040
apoA-1 + HDL-C	0.77 (0.63–0.90)	–	0.83	0.67	0.002

*AUC, area under the curve; LDL-C, low-density lipoprotein-cholesterol; HDL-C, high-density lipoprotein-cholesterol; apoA-1, apolipoprotein A-1; apoB, apolipoprotein B; TG, triglycerides; TC, total cholesterol*.

### Dynamic Alterations in Lipid Profiles and Associations With Inflammatory Indicators

**Figure 4:**
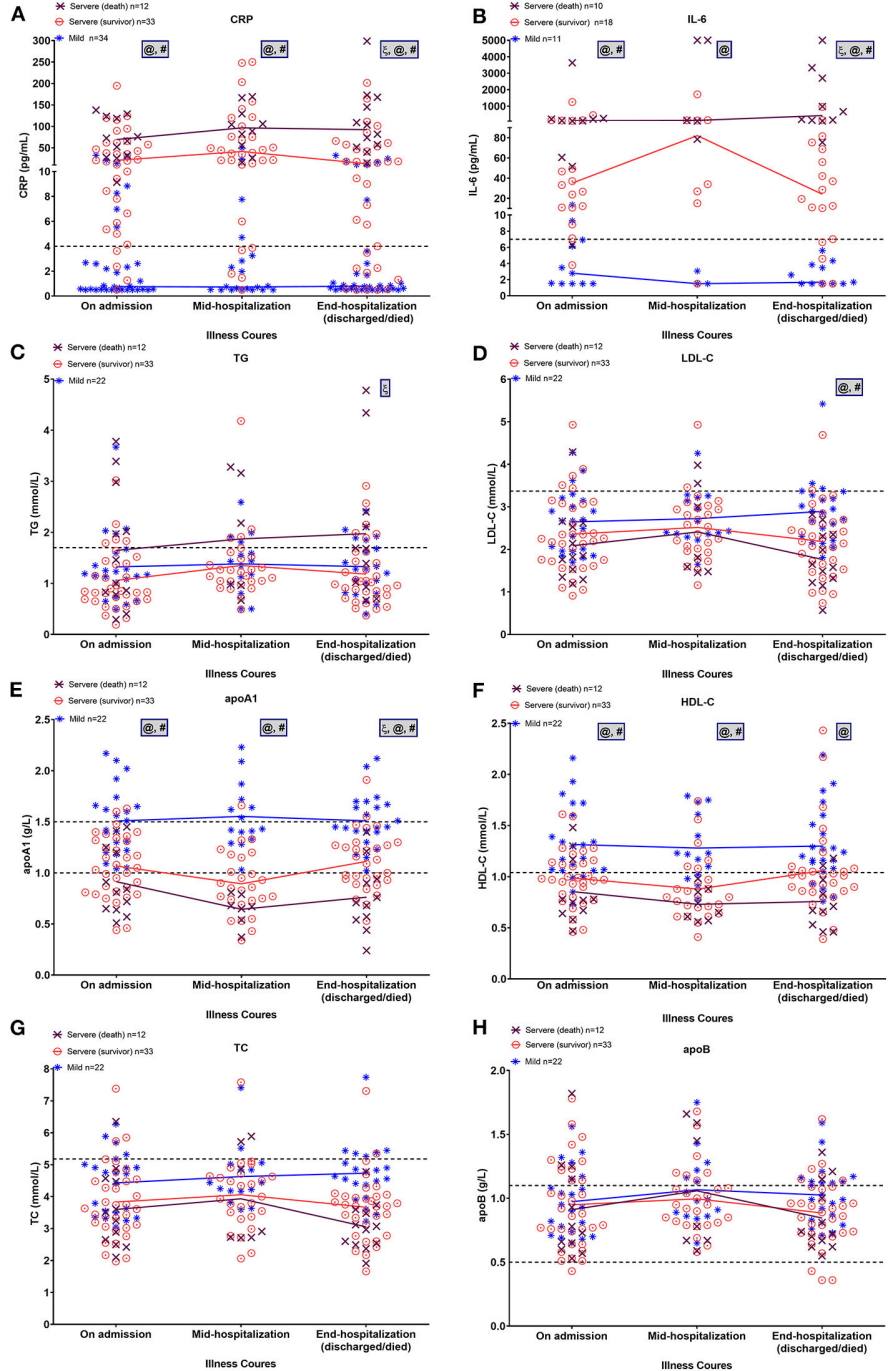
Dynamic alterations in lipid and major laboratory markers from admission in COVID-19 patients. Temporal changes in CRP **(A)**, IL-6 **(B)**, TG **(C)**, LDL-C **(D)**, apoA-1 **(E)**, HDL-C **(F)**, TC **(G)**, and apoB **(H)** in a subset of COVID-19 patients with ≥2 longitudinal data across three time periods, including on admission, mid-hospitalization, and end of hospitalization. Horizontal dashed lines indicate normal reference range of factors. Mean values of normally distributed parameters (lipid metrics) and median values of non-normally distributed factors (CRP and IL-6) in each group at three time periods are linked by lines. Significant differences among three groups at each time point were compared using one-way ANOVA with Tukey's multiple comparisons test or Kruskal-Wallis test as appropriate. Statistical significance (*p* < 0.05) is indicated by ξ between severe (death) and severe (survivor) cases, @ between severe (death) and mild cases, and # between severe (survivor) and mild cases. IL-6, interleukin-6; CRP, C-reactive protein; TG, triglycerides; TC, total cholesterol; LDL-C, low-density lipoprotein-cholesterol; HDL-C, high-density lipoprotein-cholesterol, apoA-1, apolipoprotein A-1; apoB, apolipoprotein B.

Figure 4 shows the changes in inflammatory factors and lipid profiles in the mild, severe-surviving, and severe-non-surviving groups from hospital admission, mid-term hospitalization, and end of hospitalization. As illustrated in Figures 4A,B, throughout hospitalization, CRP and IL-6 levels were significantly and continuously high in the severe-surviving and mortality cases but showed low levels among mild cases. Notably, compared with that in the severe-surviving group, both CRP and IL-6 levels in mortality cases were significantly higher at the end of hospitalization (*p* < 0.05).

On admission, regardless of severity or outcome, most patients presented comparable TG and LDL-C levels (Figures 4C,D). By the end of hospitalization, however, TG levels displayed a slight upward trend in the mortality cases and were significantly higher than that in the severe survivors (*p* = 0.013); in addition, LDL-C levels were significantly lower in severe survivors and non-survivors compared to that in the mild cases (both *p* < 0.01). Levels of apoA-1 and HDL-C were inversely proportional to disease severity, with mortality cases showing continuously lower levels across hospitalization (Figures 4E,F). Of note, after a slight downward trend in mid-term apoA-1 levels, severe survivors showed a significant recovery in apoA-1 levels at the end of hospitalization (vs. mid-term apoA-1 levels, *p* = 0.02). By the end of hospitalization, the lowest apoA-1 levels were found in severe cases with a fatal outcome (*p* < 0.01). For TC and apoB, no significant differences were observed among the three groups across the three time points (Figures 4G,H).

**Figure 5:**
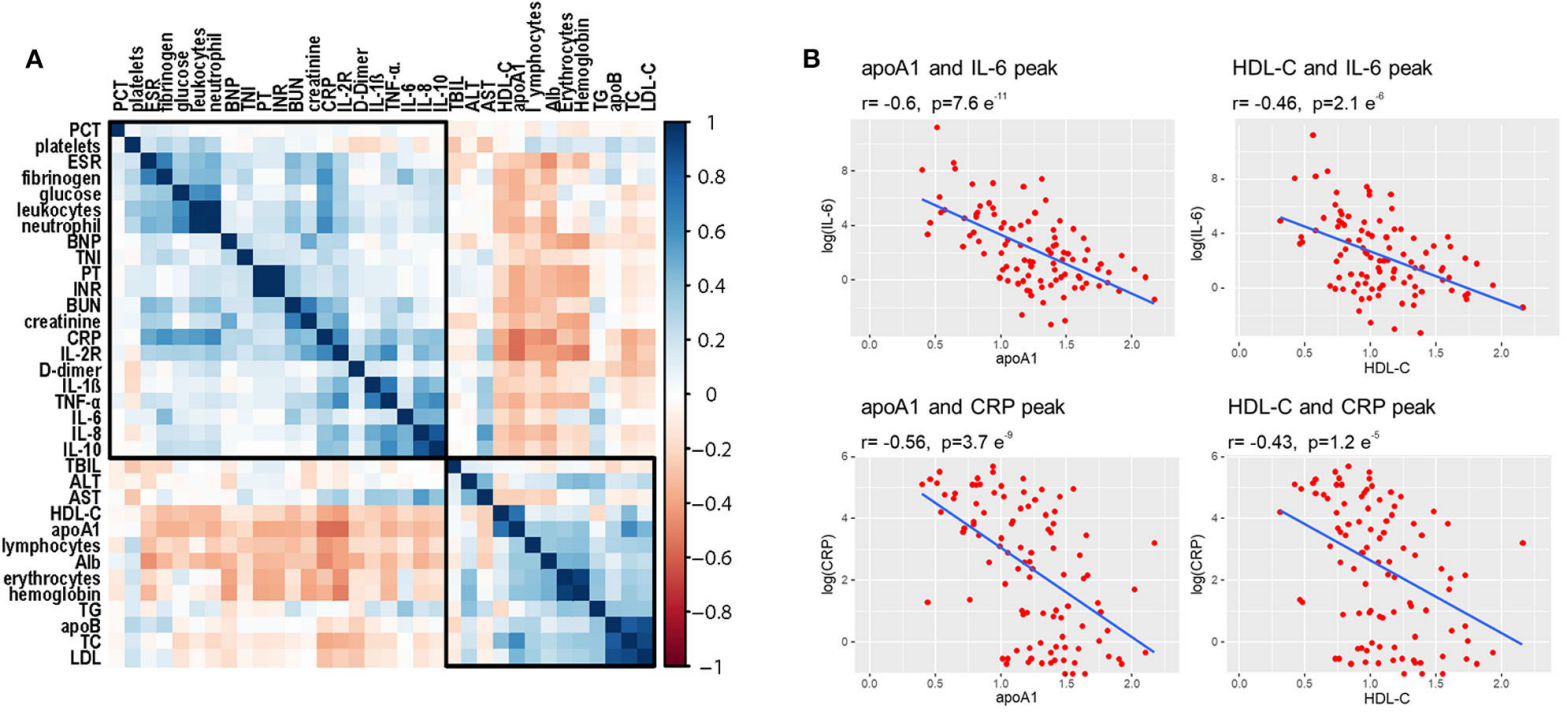
Correlations among lipid profiles and laboratory parameters. **(A)** Heatmap values represent pairwise Spearman rank correlation coefficients. Blue indicates positive correlation, red indicates negative correlation. **(B)** Spearman correlation coefficient analysis shows that initial HDL-C and apoA-1 levels were significantly inversely correlated with peak values of CRP and IL-6 during disease course.

Correlation analysis was performed to detect potential factors related to lipid characteristics. As shown in Figure 5A, admission lipid profiles, especially apoA-1 and HDL-C, were negatively correlated with inflammatory factors, such as CRP and IL-6. Admission apoA-1 and HDL-C levels were inversely correlated with peak CRP and IL-6 concentrations during the clinical course of the disease (Figure 5B).

## Discussion

Our study highlighted an important association between lipid profiles and fatal clinical outcomes in COVID patients. The main findings are as follows: (1) COVID-19 patients in severe disease were characterized by decreased apoA-1 and HDL-C levels; (2) low apoA-1 and HDL-C levels on admission were able to predict COVID-19 severity and mortality during hospitalization; and (3) apoA-1 and HDL-C levels were strongly correlated with inflammatory indicators, and deviated markedly from the normal reference range in severe cases throughout the course of the disease.

Previous studies have shown that infection and sepsis are accompanied by a metabolic change in the lipid profile, featuring hypertriglyceridemia and reduced HDL-C levels in serum (4, 8). Lipid metabolism dysregulation has also been confirmed in septic patients secondary to both community and hospital-acquired pneumonia (9, 10). In the context of COVID-19, excessive cytokine activation in response to SARS-CoV-2 infection appears to contribute to multiple organ dysfunction. As a result, sepsis and septic shock are frequently observed complications in severe COVID-19 patients (11, 12). Therefore, it is not surprising that serum apoA-1 and HDL-C levels were lower in severely ill patients, especially non-survivors, compared to mild cases.

Both apoA-1 (*r* = −0.55; *p* < 0.001) and HDL-C (*r* = −0.45; *p* < 0.001) levels were negatively related to SOFA scores, a common diagnostic tool for identifying sepsis severity (13). Based on multivariate analyses, decreased apoA-1 and HDL-C levels were independently associated with COVID-19 severity after adjusting for established indicators of severity, such as age, low albumin, and increased D-dimer, CRP, and IL-6 levels (14, 15). These covariates were included in the multivariate analysis due to their close association with sepsis development reported in previous studies (16, 17). In addition, ROC analysis illustrated that decreased apoA-1 and HDL-C levels were strong predictors of COVID-19 severity. In line with our findings, Groin et al. found that low serum HDL-C concentration on admission is a risk factor for the development of severe sepsis (18).

Our results also highlighted the predictive value of decreased HDL/apoA-1 levels on admission to in-hospital death in COVID-19 patients. Almost half of our research population developed into severe cases, with a relatively high mortality rate of 15.1%. This may be because Leishenshan Hospital was a designated hospital for treating complicated patients transferred from other local hospitals. Our study, for the first time, illustrated that in-hospital death increased significantly in patients with low serum apoA-1 (<0.95 g/L) or HDL-C (<0.84 mmol/L). In addition, ROC analysis verified the predictive value of HDL-C and apoA-1 levels for in-hospital death among COVID-19 patients. This is in agreement with previous study, which found that low apoA-1 concentration is independently associated with the 30-day mortality rate in septic patients (19). Interestingly, here, the temporal recording of lipid profiles showed that the initial decrease in apoA-1/HDL-C levels in survivors began to recover at the end of hospitalization. A similar tendency in HDL-C change has also been observed in patients recovering from sepsis (20). Here, however, apoA-1 rapidly deteriorated in non-survivors throughout the clinical course of the disease.

The underlying mechanisms of HDL-C reduction in severe COVID-19 patients and its association with increased mortality are not fully understood. HDL-C and its major structural protein (apoA-1) directly exert anti-inflammatory effects by neutralizing lipopolysaccharides (LPS), thus playing an important role in host resistance to bacterial, viral, and parasitic infection (21). The protective role of apoA-1 is also evidenced in acute lung injury and acute respiratory distress syndrome. Specifically, apoA-1-deficient mice exhibit enhanced recruitment of neutrophils and monocytes to airspace under LPS inhalation (22). However, both HDL-C and its beneficial effects can be disturbed by inflammation (23, 24). For example, pro-inflammatory cytokines like IL-6 and CRP directly inhibit apolipoprotein synthesis enzyme activity, resulting in reduced apoA-1 and HDL-C production (25). In our study, IL-6 and CRP concentrations were significantly higher in the severe group, and were negatively correlated with lipid indicators apoA-1 and HDL-C. We also found that serum amyloid A (SAA), an acute phase protein, was markedly increased in severe patients. SAA-enriched HDL is reported to clear more rapidly from circulation than normal HDL (26). Hence, the inflammatory-induced humoral innate response to scavenge lipoprotein from circulation may be another potential mechanism leading to low-HDL-C levels. As a result, a vicious cycle occurs in severely ill COVID-19 patients, with a deficiency in HDL-C resulting in cytokine overproduction and a further depletion of HDL-C.

In our study, TC and LDL-C levels in severe patients tended to follow a pattern similar to that of HDL-C. Low TC and LDL-C levels are considered as markers of malnutrition, as nutrition provides the basic substrate for cholesterol synthesis (27). Furthermore, early enteral nutrition is reported to accelerate the recovery of TC levels (20). Consistently, the nutrition states of patients deteriorated in our study, as reflected by continuously decreased levels of albumin in the severe group. Like HDL-C, inflammatory mediators also participate in impaired LDL-C synthesis. Thus, hypocholesterolemia may reflect both malnutrition and an overactive inflammatory status in severe COVID-19 patients.

Although admission TG levels were comparable between mild and severe cases, TG levels were remarkably elevated in non-survivors. Serum TG frequently increases under a septic environment due to reduced TG hydrolysis. Inflammatory cytokines also contribute to inhibit LPL activity, overproduction of free fatty acid, and TG synthesis (26). Besides, after comparing the survival rates between four groups of patients stratified by TG and apoA-1 levels, we found that patients with lower apoA-1 levels and elevated TG levels displayed the unfavorable prognosis with the lowest survival rate (Supplementary Figure 2). Thus, we considered that elevated TG levels, together with persistently low lipoprotein cholesterol concentrations, might be a marker of uncontrolled inflammation and increased risk of death in COVID-19 patients. And further assessment in larger cohorts are required for validation.

## Study Limitations

There are several limitations in our study. First, given the small sample size, to avoid overfitting, we only calculated the Kaplan-Meier survival curve to evaluate the prognostic values of apoA-1 and HDL-C but did not conduct multivariate cox regression to assess the independent prognostic values of these lipid metrics. Thus, further larger cohorts are warranted to verify our conclusions. Second, some patients were already in poor condition when transferred from the local hospital to Leishenshan Hospital, resulting in a higher rate of severe cases in our study. Further studies on outpatients and other mobile hospitals are required to provide a more complete picture of the relationship between lipid profiles and disease progression. Third, our study only focused on lipid concentrations rather than their quality. Therefore, whether lipid particle composition and functional alteration can affect COVID-19 outcomes deserves further investigation.

## Conclusions

Lipid metabolism disorders, characterized by low HDL-C and apoA-1 levels, were found in severely ill COVID-19 patients. The altered HDL-C and apoA-1 levels were negatively correlated with inflammatory indicators. Low apoA-1 and HDL-C levels on admission exhibited predictive value in discriminating disease severity and mortality during hospitalization. Our study examined COVID-19 in regard to lipid metabolism, and thus provides new insights into the disease.

## Data Availability Statement

The original contributions presented in the study are included in the article/Supplementary Materials, further inquiries can be directed to the corresponding author/s.

## Ethics Statement

The studies involving human participants were reviewed and approved by The Ethics Commission of Renji Hospital. The patients/participants provided their written informed consent to participate in this study.

## Author Contributions

JS and JP: conceived and designed the experiments. ZC, PN, HG, LS, FY, XQ, WW, MZ, XY, and YZ: collected and analyzed the data. JS, ZC, and JP: wrote the manuscript. All authors contributed to the article and approved the submitted version.

## Conflict of Interest

The authors declare that the research was conducted in the absence of any commercial or financial relationships that could be construed as a potential conflict of interest.
