# Supplementary material for: Lipid Profile Features and Their Associations With Disease Severity and Mortality in Patients With COVID-19
Source: Front Cardiovasc Med. 2020 Dec 4;7:584987. doi: 10.3389/fcvm.2020.584987 (PMC7746652; doi:10.3389/fcvm.2020.584987)
Supplement: Supplementary file 1 [file Data_Sheet_1.PDF]

Supplementary Material

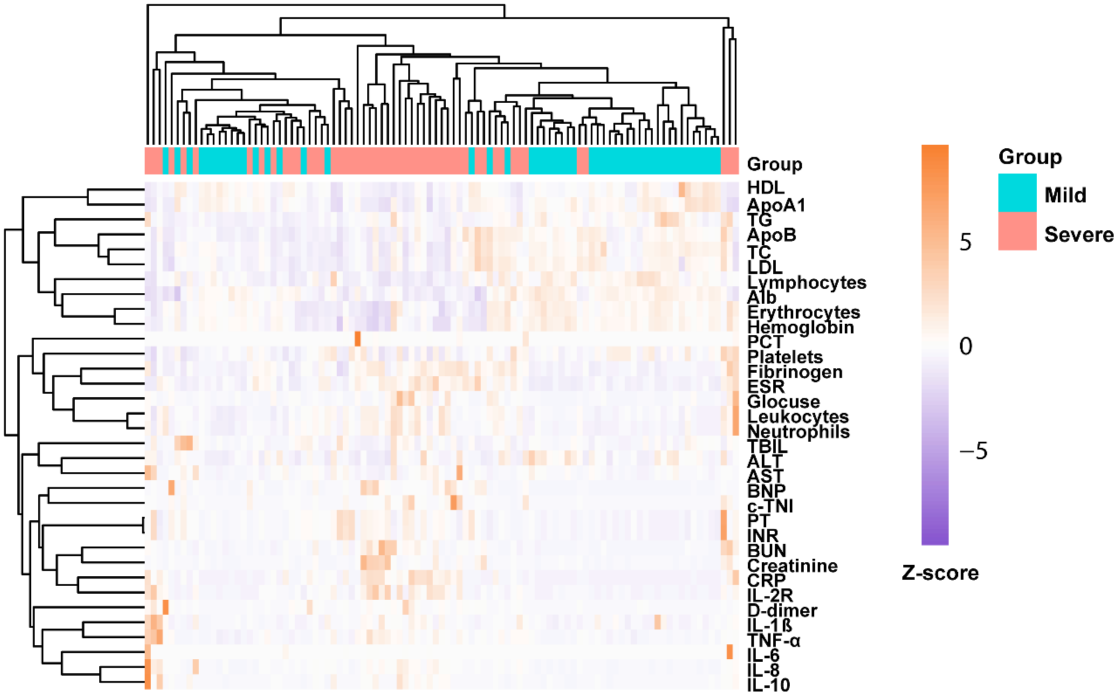

Supplementary Figure 1. Admission characteristics of laboratory parameters between mild and severe COVID-19 patients. Unsupervised hierarchical clustering of laboratory measurements clustering was applied based on laboratory parameters. Levels of laboratory metrics were scaled through calculating z-score (subtract the mean and then divide by the standard deviation of each row). The y axis represent laboratory values after z-scoring by row, and on the x axis are individual cases. Annotations show severe cases in pink and mild cases in cyan.

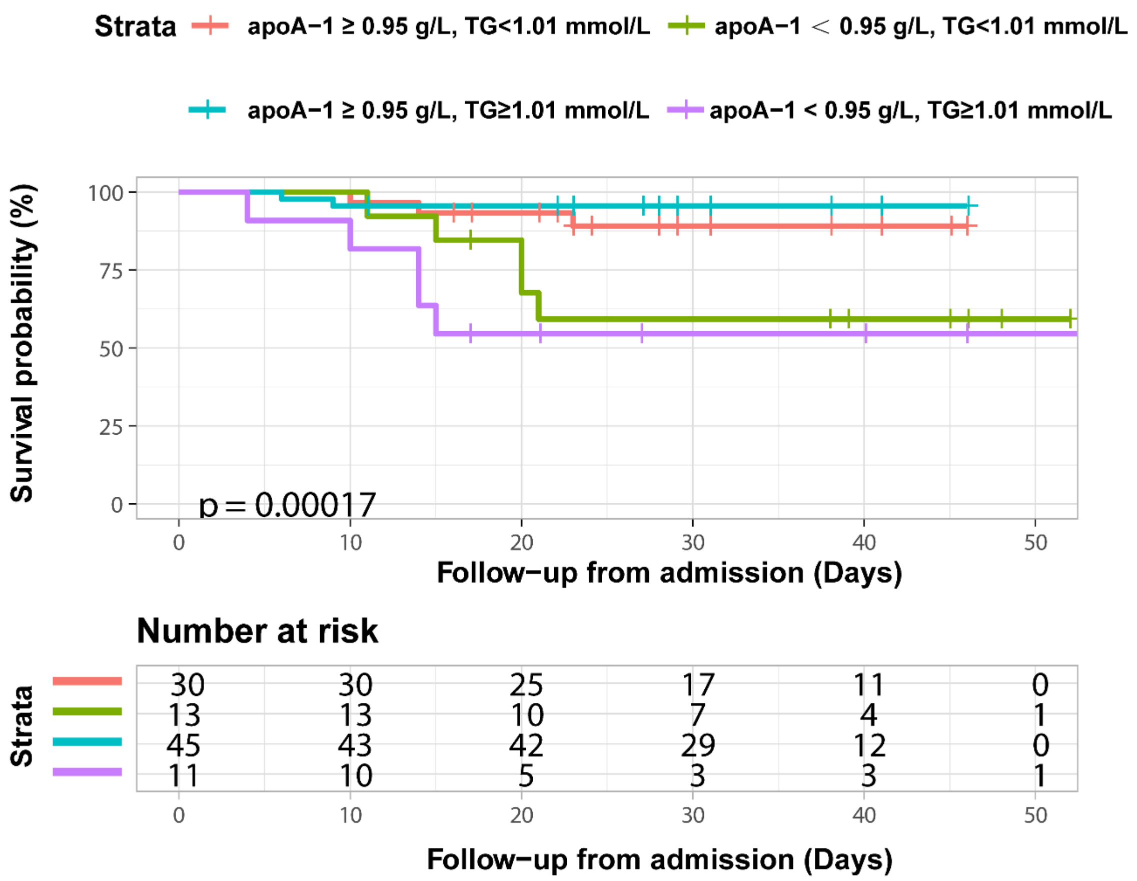

Supplementary Figure 2. Kaplan–Meier survival curves for in-hospital death based on TG and apoA-1 concentration. Kaplan–Meier curves showed that COVID-19 patients with apo A1 levels and TG levels above and below the best cut-off value had obvious disparity in terms of survival time (p<0.0001).

Lipid metrics	Adjusted OR 95%CI	p-value
**apoA1(10^{-1} g/L)**	0.642 (0.428- 0.964)	0.033
**HDL-C (10^{-1}mmol/L)**	0.675 (0.470- 0.971)	0.034
**Total cholesterol (mmol/L)**	0.640 (0.219-1.867)	0.414
**Triglyceride (mmol/L)**	0.807(0.167-3.912)	0.790
**LDL-C (mmol/L)**	1.090 (0.268-4.430)	0.903
**apoB(g/L)**	2.152 (0.042-110.054)	0.703

Supplementary Table 1. Multivariate analysis of lipid metrics and severe condition.

The multivariate analysis further adjusted for age, IL-6 levels on admission, CRP levels on admission, D-dimmer levels, SOFA-score, presence of comorbidity. OR: odds ratio.
